# CORRIGENDUM

**DOI:** 10.1002/hsr2.723

**Published:** 2022-07-21

In the article by Dadras O et al.^1^, the affiliation 1 was incomplete and the correct presentation is given below.

^1^Iranian Research Center for HIV/AIDS, Iranian Institute for Reduction of High‐Risk Behaviors, Tehran University of Medical Sciences, Tehran, Iran

Also, the Acknowledgement section has been corrected as given below.

The present study was conducted in collaboration with Khalkhal University of Medical Sciences, Iranian Research Center for HIV/AIDS, Tehran University of Medical Sciences, Tehran, Iran.

Lastly, Reference 2 has been corrected as given below.

Mehraeen E, Dadras O, Afsahi AM, et al. Vaccines for COVID‐19: A Systematic Review of Feasibility and Effectiveness. *Infect Disord Drug Targets*. 2022;22(2):e230921196758.

The online version has been updated to reflect all the above changes.

